# Utility of a simplified ultrasonography scoring system among patients with rheumatoid arthritis: A multicenter cohort study

**DOI:** 10.1097/MD.0000000000023254

**Published:** 2021-01-08

**Authors:** Yushiro Endo, Shin-ya Kawashiri, Shimpei Morimoto, Ayako Nishino, Momoko Okamoto, Sosuke Tsuji, Ayuko Takatani, Toshimasa Shimizu, Remi Sumiyoshi, Takashi Igawa, Tomohiro Koga, Naoki Iwamoto, Kunihiro Ichinose, Mami Tamai, Hideki Nakamura, Tomoki Origuchi, Yukitaka Ueki, Tamami Yoshitama, Nobutaka Eiraku, Naoki Matsuoka, Akitomo Okada, Keita Fujikawa, Hideo Otsubo, Hirokazu Takaoka, Hiroaki Hamada, Tomomi Tsuru, Shuji Nagano, Arinobu Yojiro, Toshihiko Hidaka, Yoshifumi Tada, Atsushi Kawakami

**Affiliations:** a Department of Immunology and Rheumatology, Division of Advanced Preventive Medical Sciences, Nagasaki University Graduate School of Medical Sciences; b Kyushu Multicenter Rheumatoid Arthritis Ultrasound Prospective Observational Cohort Study Group, Japan; c Innovation Platform & Office for Precision Medicine, Graduate School of Biomedical Sciences, Nagasaki University, Nagasaki, Japan.

**Keywords:** biological DMARDs, rheumatoid arthritis, simplified US scoring system, targeted synthetic DMARDs, ultrasonography

## Abstract

We aimed to evaluate the utility of a simplified ultrasonography (US) scoring system, which is desired in daily clinical practice, among patients with rheumatoid arthritis (RA) receiving biological/targeted synthetic disease-modifying antirheumatic drugs (DMARDs).

A total of 289 Japanese patients with RA who were started on tumor necrosis factor inhibitors, abatacept, tocilizumab, or Janus kinase inhibitors between June 2013 and April 2019 at one of the 15 participating rheumatology centers were reviewed. We performed US assessment of articular synovia over 22 joints among bilateral wrist and finger joints, and the 22-joint (22j)-GS and 22-joint (22j)-PD scores were evaluated as an indicator of US activity using the sum of the GS and PD scores, respectively.

The top 6 most affected joints included the bilateral wrist and second/third metacarpophalangeal joints. Therefore, 6-joint (6j)-GS and -PD scores were defined as the sum of the GS and PD scores from the 6 synovial sites over the aforementioned 6 joints, respectively. Although the 22j- or 6j-US scores were significantly correlated with DAS28-ESR or -CRP scores, the correlations were weak. Conversely, 6j-US scores were significantly and strongly correlated with 22j-US scores not only at baseline but also after therapy initiation.

Using a multicenter cohort data, our results indicated that a simplified US scoring system could be adequately tolerated during any disease course among patients with RA receiving biological/targeted synthetic DMARDs.

## 1 Introduction

Ultrasonography (US) is a noninvasive and valuable imaging tool comparable to but more accessible than magnetic resonance imaging (MRI) for joint assessment among patients with rheumatoid arthritis (RA).^[1]^ Among the different imaging tools described in the European League Against Rheumatism^[2]^ recommendations, US is especially helpful for the following various situations encountered during daily clinical practice: diagnosis of RA, evaluation of disease activity/treatment response/prognosis, and support of remission surveillance.^[3]^ Experts have recommended that patients with RA receiving disease-modifying antirheumatic drugs (DMARDs) undergo joint assessment using UA at baseline and after 3 to 6 months to assess the initial response to each therapy^[4]^ based on the treat-to-target (T2T) strategy of RA.^[5]^ However, with increasing US assessment opportunities for the tight management of RA, a more simplified US assessment strategy that maintains accuracy is desired in daily clinical practice.

The original US scoring system of RA had been developed by Naredo et al^[6]^ who had conducted US assessment on 12 joints (bilateral elbow, wrist, second/third metacarpophalangeal, knee, and ankle joints). In addition, our previous study suggested that simplified US assessment including 6 synovial sites over 6 joints (bilateral wrist and second/third metacarpophalangeal joints), as well as US assessment, including 24 synovial sites over 12 joints, reflected clinical disease activity and serum angiogenic factors.^[7]^ However, few studies have evaluated the utility of a simplified US scoring system by consecutive US assessments at multicenters.

Using a multicenter US cohort data, the present study thus consecutively evaluated the utility of a simplified US scoring system involving 6 limited synovial sites among patients with RA receiving biological/targeted synthetic DMARDs.

## 2 Methods

### 2.1 Study design

This study is part of an ongoing nonrandomized, multicenter, prospective cohort study of patients with active RA who received biological or targeted DMARD therapy at 15 participating rheumatology centers within the Kyushu region of Japan since June 2013. Here, we evaluate clinical disease activity and US findings every 3 months for a year starting from the initiation of new biological or targeted synthetic DMARDs.

The study is registered with the University Hospital Medical Information Network Clinical Trials Registry (http://www.umin.ac.jp/ctr/, #UMIN000012524) and was approved by the Institutional Review Board of Nagasaki University (Approval No. 13102866). All patients gave their signed informed consent to participate in accordance with the Helsinki declaration.

### 2.2 Patients

A total of 289 Japanese patients with RA who were started on tumor necrosis factor inhibitors (e.g., infliximab, adalimumab, etanercept, certolizumab pegol, and golimumab), abatacept, tocilizumab, or Janus kinase inhibitors (e.g., tofacitinib and baricitinib) between June 2013 and April 2019 at one of the 15 participating rheumatology centers were reviewed. All patients were required to satisfy the 1987 American College of Rheumatology and/or the 2010 American College of Rheumatology/European League Against Rheumatism criteria for RA.^[8,9]^

Biological/targeted synthetic DMARD dosages were administered as recommended by the manufacturers: infliximab (3–10 mg/kg via intravenous infusion every 8 weeks or 3–6 mg/kg via intravenous infusion every 4 weeks), adalimumab (40 mg via subcutaneous injection every 2 weeks), etanercept (50 mg via subcutaneous injection weekly), certolizumab pegol (400 mg via subcutaneous injection every 4 weeks), golimumab (50 or 100 mg via subcutaneous injection every 4 weeks), abatacept (125 mg via subcutaneous injection weekly or 500–750 mg via intravenous infusion every 4 weeks), tocilizumab (162 mg via subcutaneous injection every 2 weeks or 8 mg/kg via intravenous infusion every 4 weeks), tofacitinib (5–10 mg via daily oral administration), and baricitinib (2–4 mg via daily oral administration).

### 2.3 Clinical disease activity assessment

The clinical disease activity of RA in each patient was evaluated using the Disease Activity Score 28 (DAS28) based on erythrocyte sedimentation rate (ESR) or C-reactive protein (CRP) at baseline and 6 and 12 months after therapy initiation.

### 2.4 US assessment

At baseline and 6 and 12 months after therapy initiation, sonographers registered by the Japan College of Rheumatology (not the attending physicians) performed US assessment of articular synovia over 22 joints. Examination sites included bilateral wrist and first to fifth metacarpophalangeal and proximal interphalangeal joints. Systematic multiplanar grayscale (GS) and power Doppler (PD) joint examinations were performed using a multifrequency linear transducer (12–18.5 MHz) and one of the following scanners: Toshiba AplioXG or Aplio300, GE Logic series 7 or 8 or HITACHI HI VISION Avius, and Noblus or HI VISION Preirus. Each joint was given a GS and PD score from 0 to 3 in a semi-quantitative manner. Thereafter, the 22-joint (22j)-GS and -PD scores, which were collectively referred to as the 22j-US scores, were evaluated as an indicator of US activity using the sum of the GS and PD scores, respectively.^[10]^ Interobserver reliability was confirmed in a previous investigation.^[11]^

Among the bilateral wrist and finger joints frequently affected by RA, the 6-joint (6j)-GS and 6j-PD scores, which were collectively referred to as the 6j-US scores, were evaluated using the sum of the GS and PD scores from 6 synovial sites over the top 6 most affected joints, respectively.

### 2.5 Statistical analyses

Missing data for disease activity indicators at 6 or 12 months due to discontinuation of biological or targeted synthetic DMARDs were treated as missing values. Categorical variables were described as frequencies and quantitative variables as medians and interquartile ranges. Within-group comparisons were made using the Mann–Whitney *U* test. Correlations were assessed using Spearman correlation coefficient. All statistical analyses were performed using JMP pro 14.0 software (SAS Institute, Cary, NC).

The effect of each visit (at baseline and 6 and 12 months) on the correlation between 22j-US scores (including 22j-GS and -PD scores) and 6j-US scores (including 6j-GS and -PD scores) was examined using the following procedure with R software (ver. 3.2.3). Initially, regression lines of the 22j-US scores on the 6j-US scores were estimated for each visit. Thereafter, the difference between each visit was determined using the sum of squared residuals. These 2 steps were iterated using visit-randomized data between baseline and 6 months and between baseline and 12 months until 500 comparisons were obtained. Finally, the probability distribution of the difference under the null hypothesis was estimated from the empirical distribution obtained from the 500 visit-randomized datasets for each comparison. The *P* value was obtained as a quantile of the difference in the original dataset under the null hypothesis distribution. The effect of each visit on the correlation between DAS28-ESR and 22j-US scores and between DAS28-ESR and 6j-US scores were also examined using methods similar to those described above.

A *P* value of <.05 (2-tailed) was considered statistically significant for all analyses. GraphPad Prism version 7.0 was used to create the figure.

## 3 Results

### 3.1 Patient characteristics

Demographic and clinical characteristics of the 289 patients with RA enrolled herein are presented (see Supplementary Table). Accordingly, the median (interquartile range) age and disease duration was 66.0 (56.0–74.0) years and 52.0 (12.0–131.0) months, respectively. Moreover, 58.8% and 52.6% of the patients received concomitant methotrexate and low-dose oral glucocorticoids, respectively, while 35.3% had a history of biological/targeted synthetic DMARD use. Tumor necrosis factor inhibitors were introduced in 105 patients (infliximab, 22; adalimumab, 21; etanercept, 19; certolizumab pegol, 19; golimumab, 24), abatacept in 93, tocilizumab in 69, tofacitinib in 9, and baricitinib in 13 patients.

### 3.2 The top 6 most affected joints

**Figure 1:**
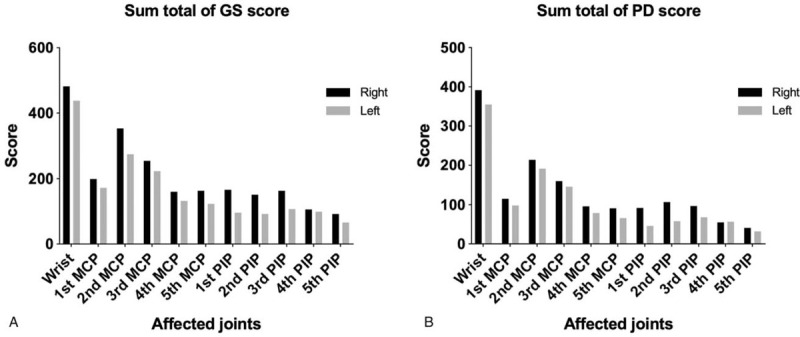
The sum total of baseline GS and PD score of patients with enrolled RA at each joint. (A) The sum total of baseline GS score of patients with enrolled RA at each joint; 482 and 438 at the wrist, 199 and 172 at the 1^st^ MCP, 353 and 274 at the 2^nd^ MCP, 254 and 222 at the 3^rd^ MCP, 160 and 132 at the 4^th^ MCP, 163 and 123 at the 5^th^ MCP, 166 and 96 at the 1^st^ PIP, 151 and 92 at the 2^nd^ PIP, 163 and 107 at the 3^rd^ PIP, 106 and 99 at the 4^th^ PIP, and 92 and 66 at the 5^th^ PIP joint each in the order of right and left side. (B) The sum total of baseline PD score of patients with enrolled RA at each joint; 392 and 355 at the wrist, 115 and 98 at the 1^st^ MCP, 214 and 191 at the 2^nd^ MCP, 160 and 146 at the 3^rd^ MCP, 96 and 79 at the 4^th^ MCP, 91 and 66 at the 5^th^ MCP, 92 and 46 at the 1^st^ PIP, 107 and 58 at the 2^nd^ PIP, 97 and 68 at the 3^rd^ PIP, 55 and 57 at the 4^th^ PIP, and 41 and 32 at the 5^th^ PIP joint each in the order of right and left side. GS = Grayscale, MCP = metacarpophalangeal, PD = power Doppler, PIP = proximal interphalangeal.

Figure 1A and B show the sum total of baseline GS and PD score of the 289 patients with enrolled RA at each joint, respectively. The top 6 most affected joints included the bilateral wrist and second/third metacarpophalangeal joints in either point of view of the sum total of GS or PD score. Therefore, 6j-GS and -PD scores were defined as the sum of the GS and PD scores from the 6 synovial sites over the aforementioned 6 joints, respectively.

### 3.3 Changes in DAS28, 22-joint US scores, and 6-joint US scores

Data for 239 and 194 patients were obtained at 6 and 12 months, respectively. Accordingly, all data regarding disease activity indicators, namely DAS28, 22j-GS and 22j-PD scores, and the 6j-GS and 6j-PD scores improved with time after therapy initiation (see Supplementary Figure).

### 3.4 Correlations among DAS28, 22-joint US scores, and 6-joint US scores

Correlations between clinical disease activity and US scores are presented in Table 1. Although both DAS28-ESR and DAS28-CRP scores were significantly positively correlated with the 22j-GS, 22j-PD, 6j-GS, and 6j-PD scores, such correlations tended to weaken with time after therapy initiation. 6j-GS and 6j-PD scores were strongly correlated with 22j-GS and 22j-PD scores, respectively. Moreover, such correlations tended to become extremely strong with time after therapy initiation.

**Table 1:** Correlations among disease activity, 22 joints US scores, and 6 joints US scores at baseline and at 6 and 12 months.

	DAS28-ESR	DAS28-CRP	22 joints-GS scores	22 joints-PD scores
22 joints-GS scores	0.43^†^0.34^†^0.26^∗^	0.43^†^0.37^†^0.28^∗^		
22 joints-PD scores	0.45^†^0.35^†^0.29^†^	0.45^†^0.43^†^0.34^†^		
6 joints-GS scores	0.42^†^, 0.35^†^0.27^∗^	0.41^†^, 0.37^†^, 0.24^∗^	0.88^†^0.90^†^0.90^†^	
6 joints-PD scores	0.38^†^, 0.36^†^, 0.30^†^	0.38^†^, 0.44^†^, 0.32^†^		0.85^†^0.94^†^0.96^†^

### 3.5 The effect of each visit on the correlation among DAS28, 22-joint US scores, and 6-joint US scores

**Figure 2:**
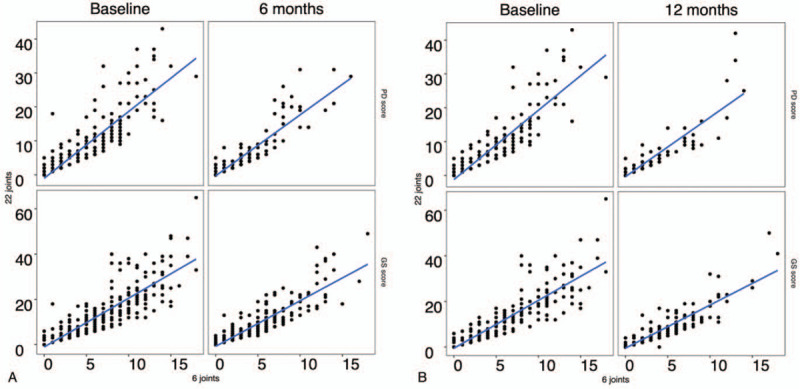
Regression lines between 22-joint and 6-joint US scores. The regression lines of the 22j-GS and -PD scores on the 6j-GS and 6j-PD scores for baseline and 6 months (A) and for baseline and 12 months (B) are shown. GS = Grayscale, PD = power Doppler, US = ultrasonography.

Figure 2 shows the regression lines of the 22j-GS and 22j-PD scores on the 6j-GS and 6j-PD scores for each visit, respectively (Fig. 2A,B). The effect of each visit on the correlation between the 22j-US and 6j-US scores was then evaluated using visit-randomized data between baseline and 6 months and between baseline and 12 months. The visitation at 6 and 12 months had a significantly greater effect on the correlation between 22j-US and 6j-US scores compared with that at baseline (not shown, *P* < .05 for all).

**Figure 3:**
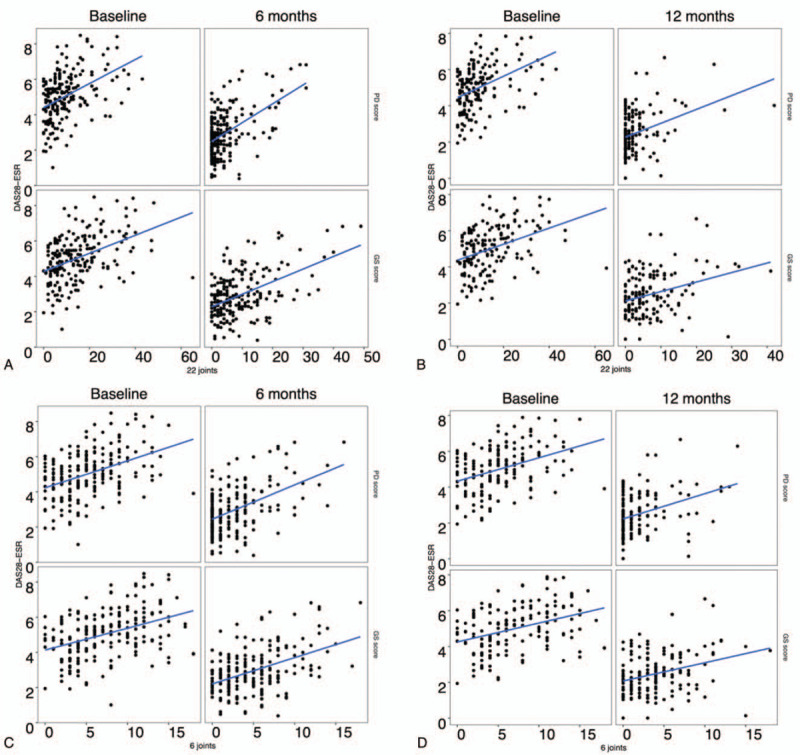
Regression lines between DAS28-ESR and US scores. The regression lines of the DAS28-ESR on the 22j-GS and -PD scores for baseline and 6 months (A) and for baseline and 12 months (B) are shown. The regression lines of the DAS28-ESR on the 6j-GS and -PD scores for baseline and 6 months (C) and for baseline and 12 months (D) are shown. ESR = erythrocyte sedimentation rate, GS = Grayscale, PD = power Doppler, US = ultrasonography.

Figure 3 shows the regression lines of the DAS28-ESR on the 22j-GS and 22j-PD scores and on the 6j-GS and 6j-PD scores for each visit (Fig. 3A–D). The visitation at 6 and 12 months had no significant effect on the correlation between DAS28-ESR and 22j-US scores and between DAS28-ESR and 6j-US scores (not shown, *P* > .05 for all).

## 4 Discussion

Although many previous studies have shown the utility of a simplified US assessment in daily practice,^[12–16]^ those studies have mentioned about patients with RA receiving conventional synthetic DMARDs or limited biological DMARDs such as antitumor necrosis factor inhibitors at single center. To the best of our knowledge, this has been the first report to evaluate the utility of a simplified US scoring system at consecutive points among patients with RA receiving whole kinds of biological/targeted synthetic DMARDs at multicenters. Although both 22j-US and 6j-US scores were significantly correlated with DAS28-ESR or -CRP scores, such correlations were weak. Conversely, 6j-US scores were significantly correlated with 22j-US scores at baseline and even stronger after therapy initiation.

Studies have shown that there is discordance between both clinical and US evaluations and that subclinical synovitis detected by US is a risk factor for flares and further bone destruction even in clinical remission.^[17,18]^ The present study showed a significant albeit weak correlation between DAS28 and both 22j-US and 6j-US scores not only at baseline but also after therapy initiation. Given the weak correlation between clinical and US evaluations, patients with RA would be recommended to undergo objective joint assessment using US, in addition to clinical assessment, after the initiation of a new therapy.

With the increased importance of US assessment, a more efficient and simplified US scoring system has been desired in daily clinical practice. Previous reports by Naredo et al^[6]^ for UA and OMERACT for MRI^[19]^ have shown that second/third metacarpophalangeal joints are considered important areas for radiographic imaging of RA. The present study showed that the bilateral wrist and second/third metacarpophalangeal joints were the most frequently affected joints among patients with RA and that 6j-US scores were significantly and strongly correlated with 22j-US scores both before and after the initiation of a new therapy. Our results suggested that a simplified US scoring system involving 6 synovial sites over 6 joints could be adequately tolerated during any disease course among patients with RA. From our results in a multicenter cohort, a simplified US scoring system may be widely tolerated in multicenter clinical trials, for example, as an indicator of US activity.

Our study has several limitations. A limitation of the present study was that the simplified US scoring system was defined using bilateral wrist and second/third metacarpophalangeal joints, which are frequently affected by RA. The present study is limited by US assessments eliminating the possible involvement of the joints other than bilateral wrist and finger joints. This simplified US scoring system may be ineffective in assessing the initial response to each therapy among patients who have no PD signal in the aforementioned six joints upon initiation of a new therapy. Second, the mode of action of tumor necrosis factor inhibitors, abatacept, tocilizumab, and Janus kinase inhibitors are different. However, we could not evaluate the utility of a simplified US scoring system for each treatment due to a small sample of each treatment. The utility of a simplified US scoring system should be distinctively evaluated by further increasing the number of subjects.

In conclusion, our results indicated that a simplified US scoring system involving 6 synovial sites over the bilateral wrist and second/third metacarpophalangeal joints could be adequately tolerated during any disease course among patients with RA receiving biological/targeted synthetic DMARDs.

## Acknowledgments

The authors wish to thank the patients and medical staff for their contributions to the study.

## Author contributions

**Conceptualization:** Yushiro Endo, Shin-ya Kawashiri.

**Data curation:** Yushiro Endo, Shin-ya Kawashiri, Ayako Nishino.

**Formal analysis:** Yushiro Endo, Shin-ya Kawashiri, Shimpei Morimoto.

**Funding acquisition:** Atsushi Kawakami.

**Investigation:** Yushiro Endo, Shin-ya Kawashiri, Ayako Nishino, Momoko Okamoto, Sosuke Tsuji, Ayuko Takatani, Toshimasa Shimizu, Remi Sumiyoshi, Tomohiro Koga, Yukitaka Ueki, Tamami Yoshitama, Nobutaka Eiraku, Naoki Matsuoka, Akitomo Okada, Keita Fujikawa, Hideo Otsubo, Hirokazu Takaoka, Hiroaki Hamada, Tomomi Tsuru, Shuji Nagano, Arinobu Yojiro, Toshihiko Hidaka, Yoshifumi Tada.

**Methodology:** Yushiro Endo, Shin-ya Kawashiri, Atsushi Kawakami.

**Project administration:** Yushiro Endo, Shin-ya Kawashiri, Atsushi Kawakami.

**Supervision:** Shin-ya Kawashiri, Atsushi Kawakami.

**Validation:** Yushiro Endo, Shin-ya Kawashiri.

**Writing – original draft:** Yushiro Endo, Shin-ya Kawashiri.

**Writing – review & editing:** Yushiro Endo, Shin-ya Kawashiri, Shimpei Morimoto, Ayako Nishino, Momoko Okamoto, Sosuke Tsuji, Ayuko Takatani, Toshimasa Shimizu, Remi Sumiyoshi, Takashi Igawa, Tomohiro Koga, Naoki Iwamoto, Kunihiro Ichinose, Mami Tamai, Hideki Nakamura, Tomoki Origuchi, Yukitaka Ueki, Tamami Yoshitama, Nobutaka Eiraku, Naoki Matsuoka, Akitomo Okada, Keita Fujikawa, Hideo Otsubo, Hirokazu Takaoka, Hiroaki Hamada, Tomomi Tsuru, Shuji Nagano, Arinobu Yojiro, Toshihiko Hidaka, Yoshifumi Tada, Atsushi Kawakami.

## Supplementary Material

Supplemental Digital Content

## Supplementary Material

Supplemental Digital Content
