# Supplementary material for: Utility of a simplified ultrasonography scoring system among patients with rheumatoid arthritis: A multicenter cohort study
Source: Medicine (Baltimore). 2021 Jan 8;100(1):e23254. doi: 10.1097/MD.0000000000023254 (PMC7793436; doi:10.1097/MD.0000000000023254)
Supplement: Supplemental Digital Content [file medi-100-e23254-s001.docx]

**Supplementary Table.** The demographic and clinical characteristics of enrolled patients with rheumatoid arthritis at baseline.

| **Variables** | **All patients (n = 289)** |
| --- | --- |
| ***Patient characteristics*** ***at baseline*** |  |
| Age (years) | 66.0 (56.0 - 74.0) |
| Disease duration (month) | 52.0 (12.0 - 131.0) |
| Male gender (%) | 63 (21.8%) |
| ***Medication*** ***at baseline*** |  |
| b/tsDMARDs-switchers (%) | 102 (35.3%) |
| Concomitant glucocorticoid use (%) | 152 (52.6%) |
| Concomitant one or more conventional DMARDs use (%) | 238 (82.4%) |
| Concomitant conventional DMARDs, number | MTX: 170, SASP: 41, TAC: 44, MZR: 7, BUC: 13, IGU: 7, CyA: 1, LEF: 1, None: 51 |
| ***Laboratory findings at baseline*** |  |
| RF positive (%) | 216 (74.7%) |
| ACPA positive (%) | 250 (86.5%) |
| ***Clinical disease activity at baseline*** |  |
| Tender joint count in 28 joints | 5.0 (2.0 - 10.0) |
| Swollen joint count in 28 joints | 4.0 (2.0 - 8.0) |
| ESR (mm/hour) | 37.0 (22.0 - 62.0) |
| CRP (mg/dl) | 0.85 (0.2 - 2.4) |
| Patient’s global assessment score (1–100-mm VAS) | 40.0 (22.0 - 65.5) |
| Physician’s global assessment score (1–100-mm VAS) | 40.0 (30.0 - 60.0) |
| DAS28-ESR | 5.0 (4.1 - 5.8) |
| DAS28-CRP | 4.2 (3.5 - 5.1) |
| SDAI | 20.8 (14.2 - 29.9) |
| CDAI | 19.5 (13.5 - 28.5) |
| ***US findings at baseline*** |  |
| 22j-GS score | 11.0 (6.0 - 20.0) |
| 22j-PD score | 6.0 (3.0 - 12.0) |
| 6j-GS score | 6.0 (4.0 - 10.0) |
| 6j-PD score | 4.0 (2.0 - 7.0) |
| ***Used b/tsDMARDs, number*** | IFX: 22, ADA: 21, ETN: 19, CZP: 19, GLM: 24, ABT: 93, TCZ: 69, TOFA: 9, BARI: 13 |

Data are reported as median (interquartile range) or number (percentage). Variables were compared using Fisher’s exact test or the Mann–Whitney U-test. b/tsDMARDs: biologic and targeted synthetic disease-modifying antirheumatic drugs; IFX: infliximab; ADA: adalimumab; ETN: etanercept; CZP: certolizumab pegol; GLM: golimumab; ABT: abatacept; TCZ: tocilizumab; TOFA: tofacitinib; BARI: baricitinib; MTX: methotrexate; SASP: salazosulufapyridine; TAC: tacrolimus; MZR: mizoribine; BUC: bucillamine; IGU: iguratimod; CyA: cyclosporin A; LEF: leflunomide; RF: rheumatoid factor; ACPA: anti-citrullinated protein antibody; DAS28: disease activity score 28; ESR: erythrocyte sedimentation rate; CRP: C-reactive protein; SDAI: simplified disease activity index; CDAI: clinical disease activity index; GS: gray scale; PD: power Doppler
