# Supplementary material for: Utility of a simplified ultrasonography scoring system among patients with rheumatoid arthritis: A multicenter cohort study
Source: Medicine (Baltimore). 2021 Jan 8;100(1):e23254. doi: 10.1097/MD.0000000000023254 (PMC7793436; doi:10.1097/MD.0000000000023254)

**Supplementary Figure.** Changes in disease activity indicators. (A) The respective median DAS28-ESR and -CRP scores decreased from 5.0 (4.1–5.8) and 4.2 (3.5–5.1) at baseline to 2.8 (2.1–3.6) and 2.1 (1.5–2.9) at 6 months and to 2.4 (1.7–3.3) and 1.8 (1.3–2.4) at 12 months, respectively. (B) The respective median 22j-GS and -PD scores decreased from 11 (6–20) and 6 (3–12) at baseline to 6 (3–12) and 2 (0–5) at 6 months and to 5 (2–9) and 0 (0–3) at 12 months. (C) The respective median 6j-GS and -PD scores decreased from 6 (4–10) and 4 (2–7) at baseline to 4 (2–7) and 1 (0–4) at 6 months and to 3 (1–6) and 0 (0–2) at 12 months.

*Abbreviations:* MCP, metacarpophalangeal; PIP, proximal interphalangeal; DAS28, Disease Activity Score 28; ESR, erythrocyte sedimentation rate; CRP, C-reactive protein; GS, gray scale; PD, power Doppler


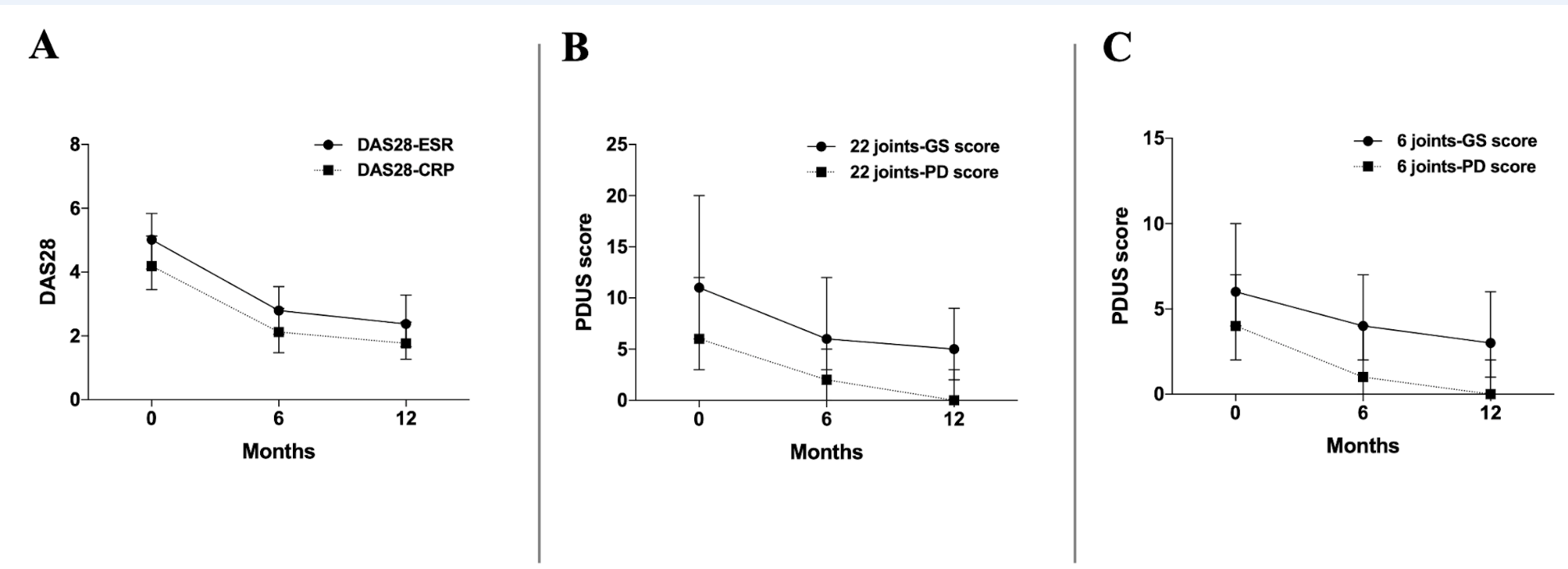

Supplement: Supplemental Digital Content [file medi-100-e23254-s002.doc]
